# Global Trends and Regional Patterns in the Epidemiology of Substance Use Disorders in 1990–2023: A Secondary Analysis of the Global Burden of Disease 2023 Study

**DOI:** 10.3390/jcm15208143

**Published:** 2026-10-20

**Authors:** Ahmed M. Ashour, Ali M. Alshahrani

**Affiliations:** 1 Department of Pharmacology and Toxicology, College of Pharmacy, Umm Al-Qura University, Makkah P.O. Box 715-21955, Saudi Arabia; 2 Department of Clinical Pharmacy, College of Pharmacy, Taif University, Taif P.O. Box 11099-21944, Saudi Arabia

**Keywords:** addiction management, health inequalities, epidemiology, public health surveillance, rehabilitation services

## Abstract

**Background:** Substance use disorders (SUDs) remain a major contributor to global morbidity and mortality, imposing substantial health, social, and economic burdens worldwide. Comprehensive assessments of long-term epidemiological trends across World Health Organization (WHO) regions remain limited. This study examined global and regional trends in the age-standardized prevalence, incidence, and mortality rates of substance use disorders between 1990 and 2023. **Methods:** This longitudinal ecological study utilized publicly available estimates from the Global Burden of Disease (GBD) 2023 Study. Annual age-standardized prevalence, incidence, and mortality rates of substance use disorders were extracted for the period of 1990–2023. Analyses were conducted for global estimates and the six WHO regions. Absolute and percentage changes between 1990 and 2023 were calculated, while temporal trends were illustrated using annual line graphs. Sensitivity analyses using five representative time points (1990, 2000, 2010, 2020, and 2023) were undertaken to assess the robustness of the observed trends. **Results:** Globally, the age-standardized prevalence rate declined from 2409.09 (95% UI: 2135.36–2704.19) per 100,000 population in 1990 to 2030.71 (95% UI: 1827.11–2260.99) in 2023, representing a 15.71% reduction. Similarly, the global age-standardized incidence rate decreased from 1052.27 (95% UI: 904.84–1212.43) to 874.07 (95% UI: 759.06–996.86) per 100,000 population, corresponding to a 16.94% decline. In contrast, the global age-standardized mortality rate increased slightly from 3.83 (95% UI: 3.44–4.24) to 3.94 (95% UI: 3.62–4.29) per 100,000 population, representing a 2.87% increase. The Region of the Americas was the only WHO region to demonstrate an increase in prevalence, rising from 3669.36 (95% UI: 3227.49–4165.00) to 3937.92 (95% UI: 3557.22–4366.84) per 100,000 population (7.32%). Mortality in this region increased from 4.46 (95% UI: 4.17–4.78) to 12.84 (95% UI: 11.28–14.62) per 100,000 population, representing a 187.79% increase, whereas incidence declined across all WHO regions. Sensitivity analyses produced findings consistent with those obtained from the complete annual dataset. **Conclusions:** Substantial regional disparities persist in the epidemiology of SUDs across the WHO regions. The marked increase in prevalence and mortality observed in the Region of the Americas contrasts with improvements in most other WHO regions, pointing to the need for region-specific prevention and treatment, as well as harm-reduction and rehabilitation strategies.

## 1. Introduction

Substance use disorders are among the leading causes of not only preventable morbidity and premature mortality but also disability and, in significant cases, mental health issues across the globe [1]. Accordingly, substance use disorders represent a major public health challenge with profound social, economic, and healthcare implications, as described by Volkow and Blanco [2]. Viewed within the provisions of the Diagnostic and Statistical Manual of Mental Disorders, substance use disorders comprise a spectrum of cognitive, behavioral, and physiological symptoms resulting from the continued use of psychoactive substances despite significant health and social consequences [3,4]. These disorders encompass dependence on substances including alcohol and opioids, cannabis and cocaine, amphetamines and sedatives, and inhalants, as well as other psychoactive substances that contribute substantially to the global burden of disease [5]. Beyond their direct health consequences, SUDs are associated with increased risks of mental illness and infectious diseases, injuries and violence, criminal justice involvement, and reduced quality of life [2,6,7]. Global monitoring initiatives underscore that substance use disorders remain a major contributor to global morbidity and premature mortality, with substantial gaps in treatment coverage globally [8,9]. Consequently, the burden of substance use extends well beyond affected individuals; as a result, it imposes considerable pressure on healthcare systems, social welfare programs, and national economies.

Globally, substance use remains an important contributor to disability-adjusted life years and premature mortality. Verifiable data from the World Health Organization and other researchers confirm that alcohol use alone accounts for millions of deaths annually and is a major risk factor for ailments from liver disease to cardiovascular disorders, injuries, and several cancers [10,11]. Similarly, opioid use disorders have contributed substantially to the escalating burden of overdose mortality observed in many regions during the past two decades, with stimulant and polysubstance use emerging as growing public health concerns in both developed and developing countries [12,13,14]. The distribution of substance use disorders is highly heterogeneous, notably with considerable variation existing across countries and regions owing to differences in socioeconomic development and other factors like demographic characteristics and healthcare infrastructure, as well as regulatory policies and cultural norms, as well as access to prevention, treatment, and rehabilitation services [15,16]. These disparities notably emphasize the need for robust comparative analyses capable of identifying populations at greatest risk and informing targeted public health interventions.

Recent studies in which researchers have relied on evidence from the Global Burden of Disease study have advanced the understanding of the changing epidemiology of substance use disorders. A recent study evaluated the global burden of substance use disorders alongside self-harm and interpersonal violence between 1990 and 2021 [17]. The authors projected future trends through 204 with their findings helping to pinpoint substantial regional variation and the continuing public health impact of these conditions. Similarly, another study examined post-COVID shifts in the global burden of substance use disorders and reported widening inequalities across countries and demographic groups [18]. Taken together, these studies demonstrate that the burden of substance use disorders has evolved considerably over the past three decades and continues to exhibit substantial variation across geographic regions.

As a source of data for this study and others that have been completed before it, the Global Burden of Disease Study, which is coordinated by the Institute for Health Metrics and Evaluation (IHME, Seattle, WA, USA), serves as an excellent source, as it provides the most comprehensive and standardized assessment of health loss worldwide [19]. Through the utilization of consistent epidemiological methods and statistical modelling techniques, the GBD Study generates annual estimates of not only incidence and prevalence but also mortality and other indicators of health status, including years lived with disability and years of life lost and even disability-adjusted life years, for hundreds of diseases and injuries across more than 200 countries and territories. The standardized methodology employed by the GBD allows for meaningful comparisons across populations and age groups, sexes and geographic regions, and levels of socioeconomic development while accounting for variations in data quality and availability [19]. Consequently, GBD estimates have become an indispensable resource for evaluating global health priorities, monitoring epidemiological transitions, and guiding evidence-based policy development.

Despite the important contributions from researchers who have relied on evidence from the GBD Study, several knowledge gaps remain. A background review of the existing literature shows that although previous studies have reported overall disease burden, there are few that have simultaneously examined multiple epidemiological indicators, including incidence, prevalence, and mortality, especially within a single comprehensive framework. Such an integrated assessment is essential because each metric captures a different dimension of disease burden and, as such, would enable a more complete understanding of the epidemiology of substance use disorders. It is also vital to indicate here that the continually updated GBD database provides an opportunity to reassess global patterns using the most recent estimates while evaluating long-term temporal changes over more than three decades. Comprehensive analyses incorporating annual observations from 1990 through 2023 are necessary and timely, as they can identify sustained increases or decreases in disease burden, detect periods of accelerated change, and quantify differences across World Health Organization regions. In addition, understanding how the burden of substance use disorders differs across the World Health Organization regions has important implications for global health planning, as identifying these patterns is essential for prioritizing public health investments and strengthening addiction management and rehabilitation services, as well as for monitoring progress toward reducing health inequalities. Such evidence is incredibly valuable for policymakers and healthcare providers as well as international organizations seeking to allocate limited resources efficiently while addressing the growing global impact of substance use disorders.

In light of these observations, the present study aimed to examine global trends and regional patterns in the burden of substance use disorders using data from the GBD 2023 Study, which provides annual estimates for the period of 1990–2023. Specifically, the study sought to describe temporal changes in the age-standardized incidence, prevalence, and mortality of substance use disorders while comparing burden estimates across the six World Health Organization regions. The study also quantified percentage changes between 1990 and 2023 to identify regional differences in the evolving burden of substance use disorders over more than three decades. The particular novelty of this study lies in that, while previous GBD evaluations focused on earlier iterations or specific substance subtypes, this one provides a long-term continuous synthesis spanning 34 years (1990–2023), utilizing the latest GBD 2023 cycle to offer updated macro-level benchmarks of post-2020 regional trajectory shifts and mortality divergence.

## 2. Methods

### 2.1. Study Design

This study employed a longitudinal ecological design using publicly available global and regional estimates from the Global Burden of Disease 2023 Study developed by the Institute for Health Metrics and Evaluation. The analysis examined temporal trends and regional patterns in the burden of substance use disorders using annual age-standardized estimates for the period of 1990–2023. The unit of analysis comprised annual global estimates and annual estimates for each of the six WHO regions.

### 2.2. Data Source

All data used in this study was obtained from the GBD Study, which provides standardized estimates of disease burden using a comprehensive methodological framework, which was accessed online via the online GBD Results Tool (https://vizhub.healthdata.org/gbd-results/ (accessed on 16 April 2026)). The data extraction was conducted in June 2026. The present study utilized annual estimates for global and the six WHO regional estimates for the period 1990–2023. Because all data are publicly available and de-identified, no participant recruitment or primary data collection was undertaken. The complete analytical data tables and regional aggregation summaries compiled in Microsoft Excel have been deposited as Supplementary Data File S1 to ensure full transparency and reproducibility of all calculations.

### 2.3. Study Variables and Definitions

The primary outcomes of interest included the age-standardized rates (per 100,000 population) of:

1. Incidence;
2. Prevalence;
3. Mortality.

Substance use disorders were defined according to the Global Burden of Disease Study classification and included both alcohol use disorders and drug use disorders. Drug use disorders comprised opioid, cannabis, cocaine, amphetamine, and other drug use disorders as defined by the GBD Study. All estimates were extracted as age-standardized rates to facilitate valid comparisons between countries with different demographic structures. Burden estimates were analyzed globally and compared across the six WHO regions. While formal temporal modeling like joinpoint regression was beyond the intended descriptive scope of this baseline macro-level evaluation, temporal trends were systematically evaluated using standardized percentage change calculations supplemented by five-year increment sensitivity checks and 95% uncertainty interval comparisons.

### 2.4. Data Extraction and Processing

Annual global and WHO regional estimates were downloaded from the GBD Results Tool for each burden indicator using the following identifiers:

○ Cause: Substance use disorders (including both alcohol and drug use disorders);
○ Measures: Incidence, prevalence, deaths;
○ Metric: Rate (per 100,000 of population);
○ Age: Age-standardized;
○ Sex: Both sexes combined;
○ Location: Global estimates and the six World Health Organization (WHO) regions (African Region, Region of the Americas, South-East Asia Region, European Region, Eastern Mediterranean Region, and Western Pacific Region);
○ Years: 1990–2023.

Individual datasets were merged using location (global or WHO region), year, and burden indicator identifiers to create the final analytical dataset.

### 2.5. Statistical Analysis

Descriptive statistics were used to summarize annual age-standardized incidence, prevalence, and mortality of substance use disorders globally and across the six World Health Organization regions. Temporal trends from 1990 to 2023 were illustrated using annual line graphs, while percentage changes between 1990 and 2023 were calculated to quantify long-term changes in disease burden. Comparative analyses were used to examine regional differences in burden across the six WHO regions using summary tables and graphical presentations. Percentage changes between 1990 and 2023 were calculated to quantify long-term changes in disease burden using the following standard formula:

$$\mathrm{Percentage}\;\mathrm{change}\;\left(\%\right)=\left(\frac{{\mathrm{Rate}}_{2023}-{\mathrm{Rate}}_{1990}}{{\mathrm{Rate}}_{1990}}\right)*100$$

where *R*_1990_ and *R*_2023_ represents the age-standardized rates in the baseline year (1990) and the final observation year (2023), respectively.

### 2.6. Sensitivity Analysis

To evaluate the robustness of the primary findings, a sensitivity analysis was conducted using eight five-year intervals (1990, 1995, 2000, 2005, 2010, 2015, 2020, and 2023) spanning the study period. The descriptive analyses, including comparisons of age-standardized incidence, prevalence, and mortality patterns, was repeated using these selected years. The findings from the sensitivity analysis were compared with those of the primary analysis based on the complete annual dataset (1990–2023) to assess the consistency of temporal and geographic patterns. These eight representative benchmark years were strategically selected to evenly span the 34-year duration, capturing decade-level epidemiological transitions without redundant annual reporting.

### 2.7. Software and Reproducibility

Data management, statistical analyses, and graphical presentations were performed using Microsoft Excel Professional Plus 2019 (Microsoft Corporation, Redmond, WA, USA).

### 2.8. Sample Size and Statistical Power

In this study, formal sample size estimation and statistical power calculations were not performed because the study included all available annual global and WHO regional estimates contained within the GBD database for the period of 1990–2023.

### 2.9. Randomization and Blinding

Randomization and blinding procedures were not applicable because the current study was wholly founded on publicly available secondary data. We, the investigators, did not recruit any participants into the study and had no influence on exposure allocation or outcome assessment.

### 2.10. Ethical Considerations

This study utilized publicly available, anonymized secondary data from the Global Burden of Disease Study. No individual participants were recruited, and no identifiable personal information was accessed. Consequently, institutional ethical approval and informed consent were not required in accordance with international guidelines for research involving publicly available secondary datasets.

## 3. Results

### 3.1. Descriptive Summary of Prevalence, Incidence, and Mortality

**Figure 1:**
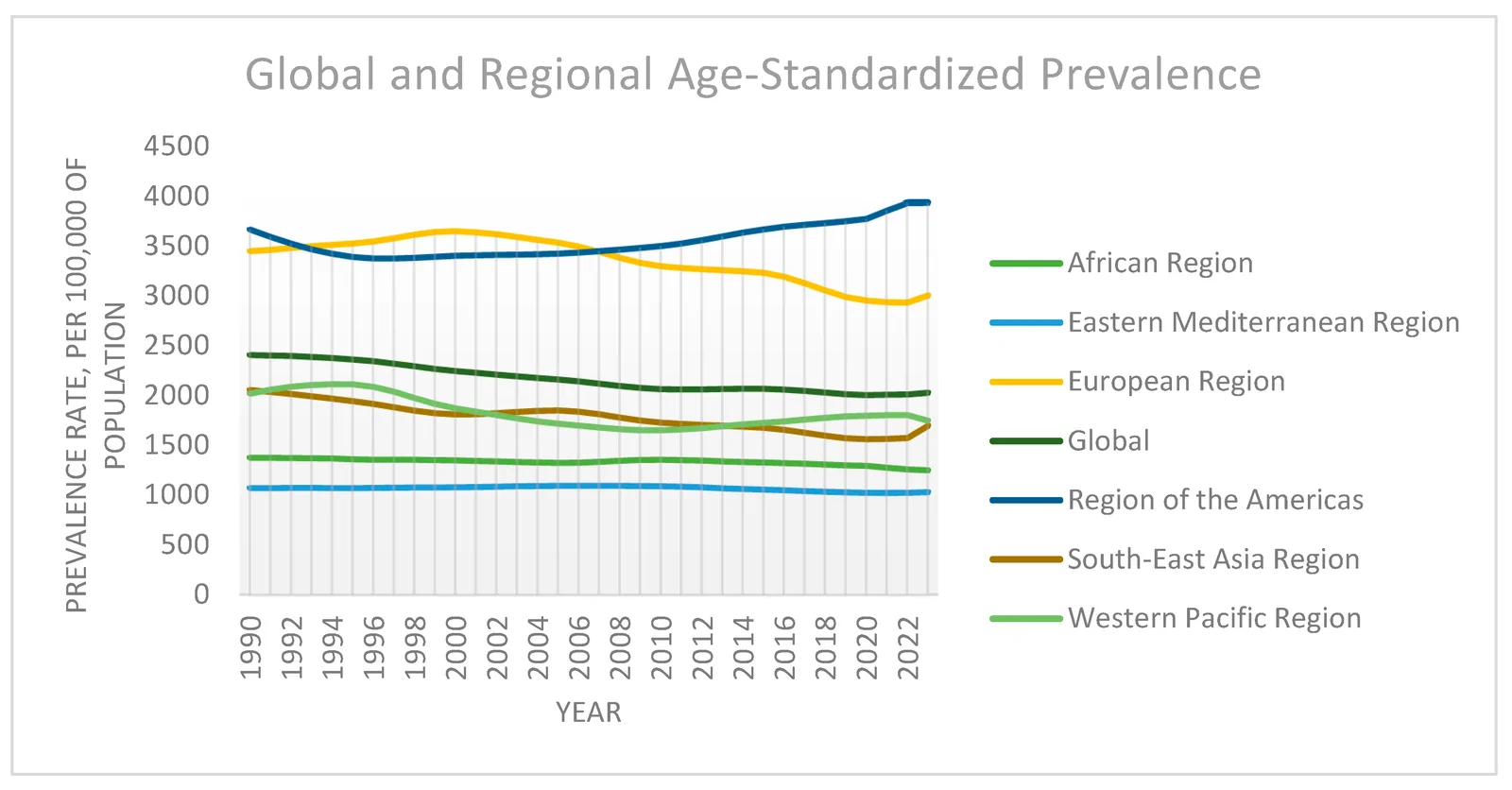
Trends in age-standardized prevalence rates of substance use disorders.

**Figure 2:**
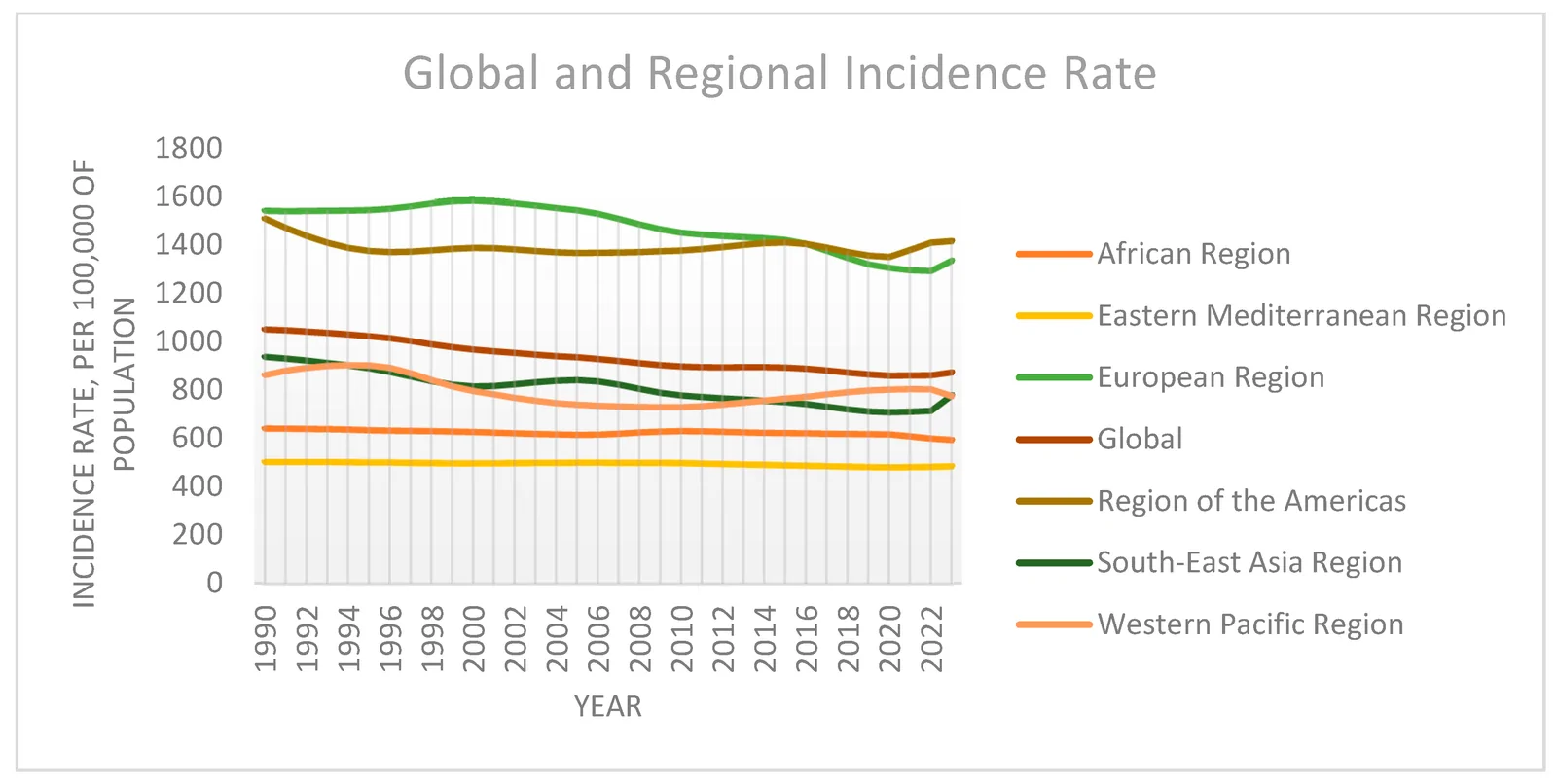
Trends in age-standardized incidence rates of substance use disorders across WHO regions, 1990–2023.

**Figure 3:**
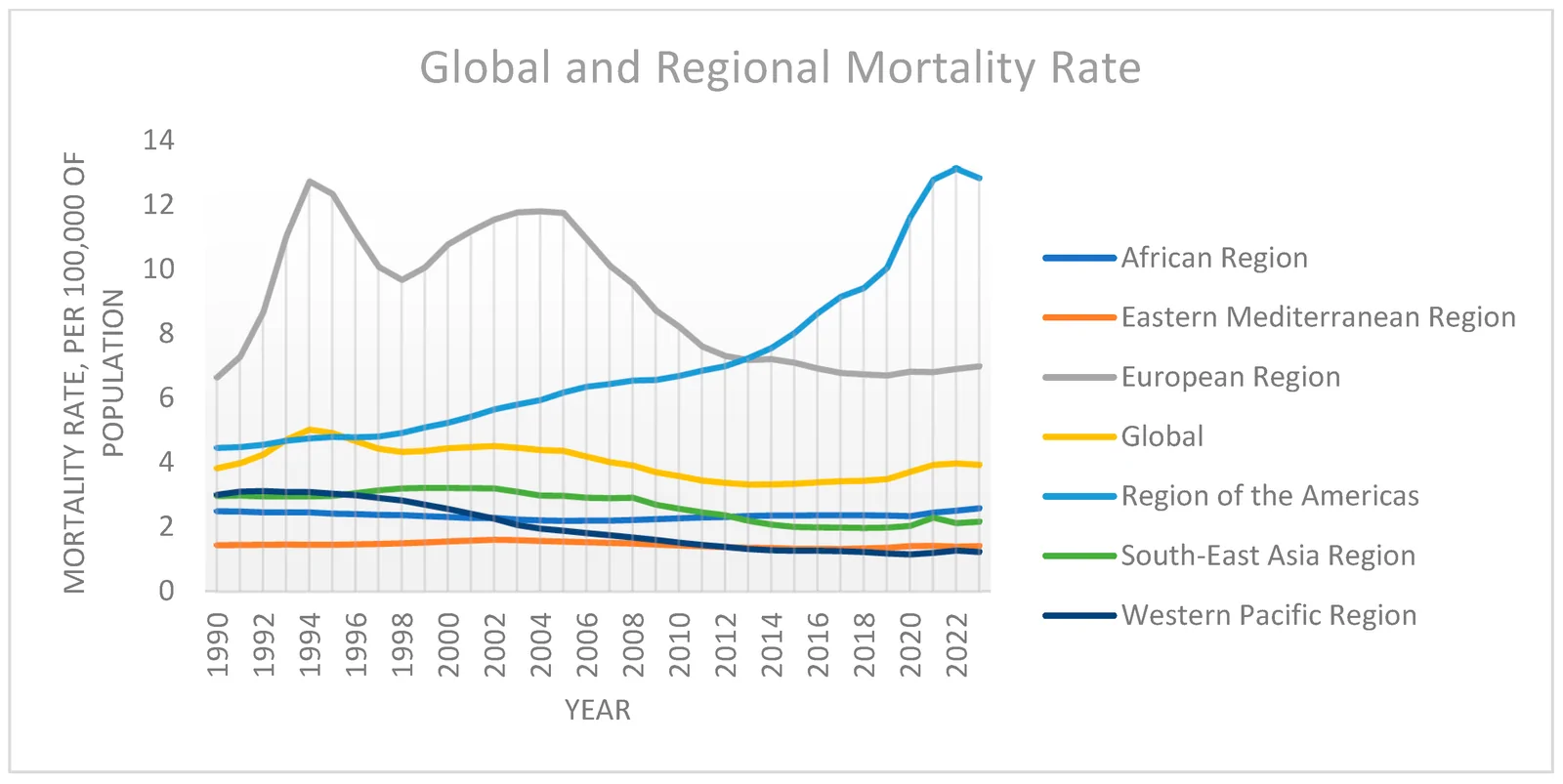
Trends in age-standardized mortality rates attributable to substance use disorders across WHO regions, 1990–2023.

Annual age-standardized prevalence, incidence, and mortality rates of substance use disorders were examined globally and across the six WHO regions between 1990 and 2023. The analyses demonstrated declining trends in both prevalence and incidence over the study period, whereas mortality showed a slight increase globally. Considerable regional heterogeneity was observed across all three epidemiological indicators. While most WHO regions experienced reductions in prevalence, incidence, and mortality, the Region of the Americas exhibited a contrasting pattern characterized by increasing prevalence and a marked rise in mortality. When evaluating regional estimates, the 95% uncertainty intervals (UIs) indicate that while overall trajectories diverged, particularly regarding the upward trend in the Region of the Americas versus steady declines elsewhere, certain regional comparisons exhibit overlapping bounds. The temporal trends and regional differences are presented in Figure 1, Figure 2 and Figure 3 and Table 1, Table 2 and Table 3.

While point estimates demonstrate clear macro-level divergence like the distinct elevation of mortality in the Region of the Americas, explicit examination of 95% uncertainty intervals reveals overlapping bounds among certain regions.

### 3.2. Trends and Patterns of Prevalence of Substance Use Disorders

Table 1 presents the age-standardized prevalence rates of substance use disorders across WHO regions between 1990 and 2023. Globally, the prevalence rate declined from 2409.09 per 100,000 population (95% UI: 2135.36–2704.19) in 1990 to 2030.71 per 100,000 population (95% UI: 1827.11–2260.99) in 2023, representing an absolute reduction of 378.37 per 100,000 population and a 15.71% decrease over the study period. Substantial regional variation was observed. The Region of the Americas recorded the highest prevalence rates in both 1990 (3669.36 per 100,000) and 2023 (3937.92 per 100,000) and was the only WHO region to experience an increase during the study period, with an absolute increase of 268.57 per 100,000 population (7.32%).

**Table 1:** Age-Standardized Prevalence Rate of SUDs per 100,000 Population, 1990 and 2023.

	1990 Value	1990 UIs	2023 Value	2023 UIs	Absolute Change	% Change
Global	2409.09	(135.36–2704.19)	2030.71	(1827.11–2260.99)	−378.37	−15.71%
African Region	1378.20	(1202.93–1562.70)	1251.87	(1112.33–1406.81)	−126.33	−9.17%
Eastern Mediterranean Region	1072.31	(944.74–1196.80)	1032.65	(920.29–1152.64)	−39.65	−3.70%
European Region	3452.66	(3082.87–3861.98)	3006.38	(2691.32–3334.57)	−446.28	−12.93%
Region of the Americas	3669.36	(3227.49–4165.00)	3937.92	(3557.22–4366.84)	268.57	7.32%
South-East Asia Region	2058.09	(1799.27–2355.64)	1698.13	(1505.36–1913.49)	−359.96	−17.49%
Western Pacific Region	2021.18	(1780.22–2266.20)	1748.95	(1539.27–1960.31)	−272.23	−13.47%

Although the European Region remained among the regions with the highest prevalence rates, it experienced a notable decline from 3452.66 to 3006.38 per 100,000 population, corresponding to a 12.93% reduction. The remaining WHO regions demonstrated declining prevalence rates between 1990 and 2023. The South-East Asia Region exhibited the largest relative reduction (17.49%), followed by the Global estimate (15.71%), the Western Pacific Region (13.47%), the European Region (12.93%), the African Region (9.17%), and the Eastern Mediterranean Region, which showed the smallest change (3.70% decrease).

### 3.3. Trends and Patterns of Incidence of Substance Use Disorders

Table 2 and Figure 2 present the age-standardized incidence rates of substance use disorders across WHO regions between 1990 and 2023. Globally, the incidence rate declined from 1052.27 per 100,000 population (95% UI: 904.84–1212.43) in 1990 to 874.07 per 100,000 population (95% UI: 759.06–996.86) in 2023, representing an absolute reduction of 178.21 per 100,000 population and a 16.94% decrease over the study period. All WHO regions experienced reductions in age-standardized incidence rates, although the magnitude of decline varied considerably. The European Region recorded the highest incidence rates in both 1990 (1542.85 per 100,000 population) and 2023 (1336.93 per 100,000 population), despite a 13.35% reduction during the study period. The Region of the Americas also exhibited consistently high incidence rates, declining from 1510.49 to 1417.61 per 100,000 population, corresponding to a 6.15% decrease.

**Table 2:** Age-Standardized Incidence Rate of SUDs per 100,000 Population, 1990 and 2023.

	1990 Value	1990 UIs	2023 Value	2023 UIs	Absolute Change	% Change
Global	1052.27	(904.84–1212.43)	874.07	(759.06–996.86)	−178.21	−16.94%
African Region	642.24	(548.00–746.27)	595.14	(510.03–686.19)	−47.11	−7.34%
Eastern Mediterranean Region	503.29	(430.11–583.97)	486.37	(422.62–557.93)	−16.92	−3.36%
European Region	1542.85	(1342.29–1753.43)	1336.93	(1172.83–1504.85)	−205.92	−13.35%
Region of the Americas	1510.49	(1280.54–1764.50)	1417.61	(1227.40–1618.41)	−92.88	−6.15%
South-East Asia Region	938.65	(779.23–1113.86)	779.78	(649.63–915.13)	−158.87	−16.93%
Western Pacific Region	863.58	(733.74–997.93)	776.21	(666.69–887.34)	−87.37	−10.12%

The greatest relative reductions in incidence were observed for the Global estimate (16.94%) and the South-East Asia Region (16.93%), followed by the European Region (13.35%), the Western Pacific Region (10.12%), the African Region (7.34%), the Region of the Americas (6.15%), and the Eastern Mediterranean Region, which demonstrated the smallest decline (3.36%). As illustrated in Figure 2, incidence rates showed a generally downward trajectory across all WHO regions throughout the study period, although the pace of decline differed between regions.

### 3.4. Trends and Patterns of Mortality of Substance Use Disorders

Table 3 the presents the age-standardized mortality rates due to substance use disorders across WHO regions between 1990 and 2023. Globally, the mortality rate increased slightly from 3.83 per 100,000 population (95% UI: 3.44–4.24) in 1990 to 3.94 per 100,000 population (95% UI: 3.62–4.29) in 2023, representing an absolute increase of 0.11 per 100,000 population and a 2.87% increase over the study period.

**Table 3:** Age-Standardized Mortality Rate of SUDs per 100,000 Population, 1990 and 2023.

	1990 Value	1990 UIs	2023 Value	2023 UIs	Absolute Change	% Change
Global	3.83	(3.44–4.24)	3.94	(3.62–4.29)	0.11	2.87%
African Region	2.49	(1.80–3.5)	2.59	(1.90–3.65)	0.09	3.69%
Eastern Mediterranean Region	1.44	(1.07–1.95)	1.41	(1.06–1.89)	−0.02	−1.69%
European Region	6.65	(6.33–7.02)	7.00	(6.68–7.36)	0.35	5.22%
Region of the Americas	4.46	(4.17–4.78)	12.84	(11.28–14.62)	8.38	187.79%
South-East Asia Region	2.97	(2.03–4.17)	2.17	(1.55–3.15)	−0.79	−26.71%
Western Pacific Region	3.01	(2.39–3.88)	1.23	(1.05–1.46)	−1.78	−59.13%

Marked regional variation was observed in mortality trends. The Region of the Americas experienced by far the largest increase, with mortality rising from 4.46 to 12.84 per 100,000 population, corresponding to an absolute increase of 8.38 per 100,000 population and a 187.79% increase between 1990 and 2023. The European Region also demonstrated a modest increase in mortality, from 6.65 to 7.00 per 100,000 population, representing a 5.22% increase. Although the European Region recorded the highest mortality rate in 1990, the Region of the Americas had surpassed all other WHO regions by 2023. In contrast, mortality rates declined in several WHO regions during the study period. The largest relative reduction was observed in the Western Pacific Region, where mortality decreased by 59.13%, followed by the South-East Asia Region (26.71%) and the Eastern Mediterranean Region (1.69%). The African Region showed a slight increase of 3.69% over the study period. Mortality trends diverged substantially across WHO regions, with the Region of the Americas exhibiting a pronounced upward trajectory whereas declining trends were observed across much of Asia and the Western Pacific (Figure 3).

### 3.5. Sensitivity Analysis Results

Sensitivity analyses based on five representative time points (1990, 1995, 2000, 2005, 2010, 2015, 2020, and 2023) demonstrated trends that were consistent with those observed using the complete annual dataset (1990–2023). The direction and magnitude of changes in prevalence, incidence, and mortality across WHO regions remained largely unchanged, supporting the robustness of the primary findings.

## 4. Discussion

The present study provides a comprehensive assessment of global and regional trends in the epidemiology of substance use disorders (SUDs) between 1990 and 2023 using standardized estimates from the Global Burden of Disease (GBD) 2023 Study. The findings demonstrate encouraging reductions in the global age-standardized prevalence and incidence rates of SUDs over the study period, which strongly suggest modest progress in reducing the occurrence of these disorders worldwide. However, these improvements were not observed uniformly across regions. Most notably, the Region of the Americas was the only World Health Organization region to experience an increase in prevalence and also exhibited a dramatic increase in mortality. These findings fundamentally emphasize the heterogeneous nature of the global SUD epidemic and highlight the importance of interpreting global averages alongside regional patterns [20]. The observed disparities are an indication that progress achieved in some regions has been offset by worsening outcomes in others, emphasizing the need for region-specific prevention, treatment, and rehabilitation strategies rather than a uniform global approach.

The declining trends in global prevalence and incidence observed in this study are broadly consistent with previous analyses of GBD data, which have reported gradual improvements in several indicators of substance use disorder burden over recent decades despite substantial regional variation. An et al. similarly reported changing global patterns in substance use disorders between 1990 and 2021, while Yu et al. highlighted considerable geographic inequalities that widened following the COVID-19 pandemic [17,18]. The present study extends these observations by demonstrating that although incidence declined across all six WHO regions, the magnitude of improvement differed substantially. The greatest reductions were observed globally and within the South-East Asia Region, whereas the Eastern Mediterranean Region experienced comparatively modest declines. These differences notably reflect variations in national public health policies, prevention initiatives, socioeconomic conditions, healthcare infrastructure, and access to evidence-based addiction treatment and rehabilitation services. Importantly, the simultaneous decline in incidence but increase in prevalence within the Region of the Americas suggests that epidemiological dynamics are influenced by factors beyond the occurrence of new cases alone. Improvements in survival, prolonged disease duration, chronic relapse patterns, and increased retention of individuals living with substance use disorders may all contribute to higher prevalence despite declining incidence. Although these mechanisms cannot be confirmed using ecological data, they provide plausible explanations that warrant further investigation.

A highly striking finding of this study was the substantial increase in age-standardized mortality observed in the Region of the Americas, where mortality increased by nearly 188% between 1990 and 2023. This increase contrasts sharply with the declining mortality observed in the Western Pacific and South-East Asia regions. It largely explains the slight increase in global mortality despite reductions in incidence and prevalence. The observed pattern is consistent with international reports like Jiang et al., who documented an escalating opioid overdose crisis, increasing availability of highly potent synthetic opioids such as fentanyl, and growing polysubstance use in several countries within the Americas [21]. Although the GBD data do not permit identification of the specific substances responsible for these trends, the findings are compatible with broader evidence indicating substantial increases in opioid-related deaths over the past decade. These observations emphasize that reducing the incidence of substance use disorders alone is insufficient if individuals living with these conditions continue to experience elevated risks of fatal overdose and other substance-related complications. Consequently, strengthening harm-reduction programs, expanding access to medication-assisted treatment, improving mental health services, and increasing the availability of rehabilitation and recovery support services remain essential components of comprehensive addiction management strategies.

### 4.1. Strengths and Limitations

This study has several important strengths, such as the utilization of standardized estimates generated through the GBD 2023 Study, thereby facilitating consistent comparisons across WHO regions over more than three decades using internationally recognized epidemiological methods. The inclusion of annual data spanning 1990 to 2023 enabled the assessment of long-term trends while minimizing inconsistencies arising from changes in surveillance systems and disease definitions. Nevertheless, several limitations should be acknowledged. First, the study relied on secondary ecological data and therefore cannot establish causal relationships or account for individual-level risk factors. Second, GBD estimates are generated using statistical modeling and may be influenced by data availability and reporting quality, particularly in regions with limited surveillance infrastructure. Third, the analysis was conducted at the regional level, which may mask important variations between individual countries within each WHO region. Lastly, the analysis relied on macro-level descriptive and percentage change evaluations over the 34-year period rather than advanced inflection-point modeling such as joinpoint regression, meaning that specific temporal shifts or year-to-year acceleration points were not formally isolated and future research work could explore this further. Furthermore, we focused on aggregate global and regional macro-level burdens in the analysis and not on demographic factors like age and sex or individual substance categories, a deliberate design choice intended to capture broad epidemiological trajectories that can be explored in dedicated sub-analyses. Due to the ecological design of this study, the findings reflect aggregate population-level trends and are subject to ecological fallacy; individual-level risks and causal mechanisms cannot be inferred directly from these data. Additionally, regional aggregation bias may obscure substantial intra-regional heterogeneity, as national and sub-national trends within a single WHO region may diverge considerably from the overarching regional average. Furthermore, because our analysis is aggregated at the WHO regional level, intra-regional heterogeneity and country-specific variations cannot be resolved. Consequently, discussions addressing potential influences such as treatment access, policy shifts, or healthcare system factors represent broader contextual hypotheses from existing literature rather than measured variables or causal determinations from this ecological study.

### 4.2. Implications

Despite these limitations, the study provides valuable evidence describing long-term changes in the epidemiology of substance use disorders worldwide. Importantly, the findings highlight persistent regional inequalities and reinforce the need for targeted public health policies, sustained investment in addiction prevention and rehabilitation services, and strengthened international collaboration to reduce the global burden of substance use disorders. More particularly, the region of the Americas requires special attention from relevant stakeholders in an effort to bring it to same progressive milestones that other WHO regions and the world at large have achieved for substance use disorders. While the rapid surge in mortality across the Region of the Americas aligns temporally with reported shifts in illicit drug supply chains and synthetic opioid expansion, these potential drivers were not directly evaluated within our dataset and should be interpreted as contextual background supported by external literature rather than definitive causal inferences. Accordingly, discussions regarding the role of illicit fentanyl, the changing patterns of healthcare and treatment access, and survival effects must be viewed as hypotheses that offer potential context for the observed mortality trends rather than empirical conclusions supported directly by our descriptive data. The rapid surge in mortality across the Region of the Americas represents a prominent observed trajectory and while an important finding, discussions regarding potential contributing drivers including synthetic opioid proliferation and shifting polysubstance use dynamics are presented strictly as contextual hypotheses from external literature rather than empirical conclusions directly evaluated within this aggregate analysis.

## 5. Conclusions

Although the global prevalence and incidence of substance use disorders has declined over the period between 1990 and 2023, there remain substantial regional disparities that warrant the attention of policymakers and other stakeholders within the healthcare sector, both regionally and globally. The marked increase in prevalence and mortality observed in the Region of the Americas contrasts with improvements in most other WHO regions and highlights the need for region-specific prevention, treatment, harm-reduction, and rehabilitation strategies. Continued surveillance of epidemiological trends is essential to inform evidence-based policies aimed at reducing the global burden of substance use disorders.

## Data Availability

The datasets analyzed in the current study are publicly available from the Global Burden of Disease (GBD) data collected by the Institute for Health Metrics and Evaluation (IHME). The study utilized country-level age-standardized estimates of prevalence, incidence, and mortality values for substance use disorders for the time period of 1990 to 2023 (global and for the six WHO regions). No new individual-level data were generated or analyzed in the current study. The analytical dataset and additional details regarding variable extraction are available from the corresponding author upon reasonable request.

## Supplementary Materials

The following supporting information can be downloaded at: https://www.mdpi.com/article/10.3390/jcm15208143/s1, Supplementary Data File S1_IHME-GBD_2023_DATA-fb9fef78-1.
